# Successful Control of Massive Bleeding in a Child with Burkitt's Lymphoma via a Biosimilar Recombinant Activated Factor VII (AryoSeven*™*)

**DOI:** 10.1155/2016/1295092

**Published:** 2016-07-10

**Authors:** Kourosh Goudarzi Pour, Fatemeh Malek, Peyman Eshghi

**Affiliations:** Paediatric Congenital Hematologic Disorders Research Center, Shahid Beheshti University of Medical Sciences, Tehran 15514-15468, Iran

## Abstract

We describe a case of a 4-year-old girl with Burkitt's lymphoma, who suffered from a massive gastrointestinal hemorrhage 3 days after chemotherapy. In spite of applying the common practice in correction of coagulopathy, thrombocytopenia persisted and bleeding became life-threatening. In the present case report, we report a successful control of bleeding with a single-dose administration of a biosimilar recombinant activated human factor VII (AryoSeven).

## 1. Introduction

Recombinant activated human factor VII (rFVIIa) is a hemostatic agent principally licensed to treat bleeding episodes and perioperative management in hemophilia A or B adults and children with inhibitors and adults with acquired hemophilia, congenital factor VII (FVII) deficiency, and Glanzmann's thrombasthenia. These indications may be presented only in a small population. rFVIIa has been widely used in off-label indications of managing bleeding episodes. The leading use of off-label indications includes managing patients suffering from coagulopathies, for example, massive, uncontrollable, or sometimes intractable hemorrhage in trauma patients [1].

rFVIIa has been found to enhance the thrombin generation on preactivated platelets. Therefore, it is assumed that the use of rFVIIa can be beneficial in providing hemostasis in other situations characterized by massive bleedings and impaired thrombin generation [2]. AryoSeven is an rFVIIa recently manufactured via recombinant technology in Iran by AryoGen pharmed. It has been proved similar to its originator medicine, NovoSeven® [3].

Notwithstanding the significant increase in pediatric case series, to date the bulk of literature on off-label use of rFVIIa has been confined to adult population. In the current study, we report a Burkitt's lymphoma case suffering from a massive GI bleeding which was controlled with a single administration of AryoSeven following the unresponsive transfusion of appropriate blood products.

## 2. Case Report

A 4-year-old girl was referred to our center with presentation of abdominal pain. Diagnostic sonography revealed an intussusception in this patient. Surgery was performed and a large mass was seen in ileocecal area. After the mass biopsy, Burkitt's lymphoma stage IIb was diagnosed since other organs were not involved.

Chemotherapy was performed in accordance with the intermediate risk group protocol named LMB89. The first course started with one dose of COP (CPM 300 mg/m^2^ vincristine 1 mg/m^2^ and 60 mg/m^2^ prednisolone; prednisolone was continued for 7 days). One week later, the second course was administered with COPADM1 (CPM IV 250 mg/m^2^/dose, MTX 3000 mg/m^2^, doxorubicin 60 mg/m^2^, Vincristine 2 mg/m^2^, and 60 mg/m^2^ Prednisolone; prednisolone was continued for 5 days). Three days after the second chemotherapy course, the patient experienced febrile neutropenia. In spite of appropriate antibiotic therapy ceftazidime 800 mg IV, vancomycin 250 mg IV, and fluconazole 50 mg were administered with supportive therapy. The patient deteriorated and suffered from massive lower GI bleeding. Her blood test is described in Table 1. Since our patient suffered from Burkitt's lymphoma, she did not experience b symptoms.

The patient received several units of pack cell, 0.25 unit/kg platelets, 15 cc/kg fresh frozen plasma, and some doses of cryoprecipitate for more than 3 days which is explained in Table 2. Different abdominopelvic sonographies, which were complemented with CT scan, showed pancolitis. Treatment was administrated with octreotide at a dose of 1 *μ*g/kg/h. It is worth mentioning that octreotide administration in this situation counts as an off-label indication but, because of its perfect efficacy at esophageal bleeding, we used it for second-line therapy, but no clinical response was observed.

After 72 hours of refractory and sever bleeding, the patient was switched to 90 *μ*g/kg injection of AryoSeven. Bleeding was successfully controlled 1 hour after administration of a single dose of AryoSeven. Vital signs were stabilized. The lowest haemoglobin concentration at the time of blood haemostasis was 6 g/dL and no more decrease in hemoglobin concentration was observed. The patient received 10 cc/kg pack cell (PC) two times as a result of the latest severe bleeding and low hemoglobin. Patient's PT, PTT, and INR are declared in Table 3.

In order to identify the source of bleeding, the patient underwent endoscopy and colonoscopy after her general condition was stabilized. No specific finding, except for a severe mucosal fragility, was found. Her last lab data are presented in Table 4, 12 hours after administration of AryoSeven.

Patient's chemotherapy was continued, her primary disease was at remission, and there was no sign of GI bleeding ever since. For other chemotherapy cycles, GCSF administration was performed to prevent febrile neutropenia. The levels of fibrinogen were not evaluated during the study because the required equipment was not available.

## 3. Discussion

AryoSeven is activated coagulation recombinant factor VII which is produced by AryoGen Pharmed. The biosimilarity of AryoSeven with NovoSeven has been approved according to the randomized, multicenter, double-blind clinical trial [4]. The usual dosage for recombinant factor VII injection is 90–120 *μ*g/kg, but it is completely dependent on patient's clinical situation. Since the clearance of this medication is higher in pediatrics, choosing the median dose (90 *μ*g/kg) was intelligent. Critically ill children with malignancies are prone to various hard to control situations. Bleeding is one of these situations that can be in many cases refractory, intractable, and life-threatening. These situations are important causes of morbidity and mortality. It is truly critical to perform the appropriate task to manage hemorrhage in patients with thrombocytopenia and haematologic malignancies. First-line strategy should be considered as appropriate blood transfusion and locating the source of bleeding. Critically ill patients may be at more risk and even haemodynamically too unstable to endure surgical or diagnostic procedures. If platelets and FFP transfusion are not successful in controlling severe GI bleeding, rFVIIa and octreotide administration have been shown to be effective alternative treatment options [5–7].

The mechanism of rFVIIa is generation of thrombin and platelet, activation of factor X, and formation of haemostatic plug [8]. There are established dose ranges in labeled and unlabeled rFVIIa administration. Currently, there is no universally accepted standard on monitoring the effect of rFVIIa. European consensus recommends that the efficacy be monitored visually and according to reductions in transfusion requirement [8]. Apparently, there are increasing calls for investigations on establishing a reliable and trustworthy guideline in off-label use of rFVIIa, which can reduce mortality [9].

Lee et al. described 3 cases of pediatric oncology patients with severe GI bleeding with no sufficient response to blood transfusions, in which rFVIIa was administered with dose range of 88–102 mcg/kg/dose. They reported that the use of rFVIIa resulted in a reduction in blood product support requirement and administration of rFVIIa was effective in 2 out of 3 patients in stopping the bleeding [5]. Our patient received octreotide prior to AryoSeven after having no response to first-line treatment such as platelet and FFP transfusion. The patient's response to AryoSeven administration was rapid and acceptable. The major bleeding was successfully stopped and there were no more decrease in Hb which might be an indicator of possible microvascular bleeding.

In conclusion, if there is no response to supportive therapy and administration of octreotide in controlling bleeding, use of recombinant factor VIIa can be lifesaving.

## 4. Conclusion

According to wide range of off-label indications of recombinant factor VIIa, we can say that it could be a wise option at refractory bleedings as the second-line therapy. The important problem is dose modification at off-label situations because there are some reports of thromboembolic events in off-label indications which were not fatal and not life-threatening.

A randomized clinical trial for off-label indication of recombinant FVIIa in pediatric hemorrhage could be helpful in assessing the safety and efficacy of this medication.

## Figures and Tables

**Table 1:** Provides lab data at time of bleeding.

Lab data	At the time of bleeding
WBC	500 mm^3^
Hb	8 g/dL
Plt	12000/mm^3^
PT	15 seconds
aPTT	46 seconds
FDP and D-Dimer^*∗*^	Normal

^*∗*^Fibrinogen level was not checked.

**Table 2:** Different blood product injection and vital sings during 3 days of bleeding.

Blood product	Unit	Body temperature before injection	Blood pressure before injection	Pulse before injection	Breath before injection
PC	1 U	37		88	22
Cryoprecipitate	—	—	—	—	—
FFP	170 mL	37		88	22
PC	4 U	38	126/48	124	25
FFP	170 mL	37.5	140/98	96	32
FFP	170 mL	37			
PC	4 U	37	90/50	123	12
PC	3 U	37	110/60	110	24
FFP	170 mL	—	—	—	—
PC	3 U	37	90/50	122	12
PC	3 U	38	90/50	120	12
FFP	170 mL	37.3	90/50	108	30
PC	3 U	37	90/50	120	32
PC	3 U	37	90/50	—	—
FFP	200 mL	37	100/60	134	28

**Table 3:** Coagulating factor evaluation during bleeding and treatment.

Date	PT patient	PT control	PT activity	INR	PTT
One day before bleeding	12	12	100	1	31
First day of bleeding	12	12	100	1	30
Second day of bleeding	13.8	12	—	1.3	28
Third day of bleeding	12	12	—	1	29

**Table 4:** Lab data 12 hours after administration of AryoSeven.

Lab data	12 hours after AryoSeven*™* injection
WBC	3880/mm^3^
Hb	11.7 g/dL
Plt	65000/mm^3^
PT	12 seconds
PTT	34 seconds
FDP and D-Dimer	Normal

## References

[1] Logan A. C., Goodnough L. T. (2010). Recombinant factor VIIa: an assessment of evidence regarding its efficacy and safety in the off-label setting. *Hematology*.

[2] Hedner U. (2006). Mechanism of action of factor VIIa in the treatment of coagulopathies. *Seminars in Thrombosis and Hemostasis*.

[3] Faranoush M., Abolghasemi H., Toogeh G. (2015). A comparison between recombinant activated factor VII (Aryoseven) and Novoseven in patients with congenital factor VII deficiency. *Clinical and Applied Thrombosis/Hemostasis*.

[4] Faranoush M., Abolghasemi H., Mahboudi F. (2016). A comparison of efficacy between recombinant activated factor VII (Aryoseven) and Novoseven in patients with hereditary FVIII deficiency with inhibitor. *Clinical and Applied Thrombosis/Hemostasis*.

[5] Lee J. H., Ng H. J., Chan M. Y. (2010). Use of recombinant factor VIIa in gastrointestinal bleeding in children with malignancies: a case series. *Proceedings of Singapore Healthcare*.

[6] Jen H., Shew S. (2008). Recombinant activated factor VII use in critically ill infants with active hemorrhage. *Journal of Pediatric Surgery*.

[7] Cello J. P., Chan M. F. (1993). Octreotide therapy for variceal hemorrhage. *Digestion*.

[8] Vincent J.-L., Rossaint R., Riou B., Ozier Y., Zideman D., Spahn D. R. (2006). Recommendations on the use of recombinant activated factor VII as an adjunctive treatment for massive bleeding—a European perspective. *Critical Care*.

[9] Brenner B., Hoffman R., Balashov D., Shutluko E., Čulić S., Nizamoutdinova E. (2005). Control of bleeding caused by thrombocytopenia associated with hematologic malignancy: an audit of the clinical use of recombinant activated factor VII. *Clinical and Applied Thrombosis/Hemostasis*.

